# Supplementary material for: WordCommentsAnalyzer: A windows software tool for qualitative research
Source: F1000Res. 2018 Sep 4;7:536. Originally published 2018 May 3. [Version 2] doi: 10.12688/f1000research.14819.2 (PMC6137411; doi:10.12688/f1000research.14819.2)
Supplement: Supplementary file 3 [file f1000research-7-17614-s0002.tgz › b2a79496-4ada-47c4-9682-a99ce867836d.zip!/Eight Iranian Health Journals/Yafteh _37.docx]

© Yafteh  . This work is licensed under CC BY 4.0 (https://creativecommons.org/licenses/by/4.0/).

Volume & Issue: دوره 11 - شماره 4

Title: بررسی فراوانی و پیش آگهی تومور کار سینوئید آپاندیس در آپاند کتومی های انجام شده در بیمارستان شهدای عشایر خرم آباد طی سالهای 1384-1379

http://yafte.lums.ac.ir/article-1-174-fa.html

تومور کار سینوئید آپاندیس نادر می باشد و شایعترین محل آن در دستگاه گوارش به ترتیب آپاندیس، روده باریک ور کتوماست. کار سینوئیدهای گوارشی غالباً بدون علانتند ودر هنگام لاپاراتومی یا همراه با التهاب آپاندیس کشف می شوند. هدف از مطالعه حاضر تعیین فراوانی تومور کارسنیوئید در آپاند کتومی های انجام شده در بیمارستان شهدای عشایر و بررسی پیش آگهی این تومور در یک دورۀ سه تا هشت ساله می باشد. مواد و روش ها: پژوهش حاضر یک مطالعه cross- sectional است که بر روی 2845 بیمار آپاند کتومی شده در بیمارستان شهدای عشایر خرم آباد در سالهای 1384-1379 انجام گرفته است. اطلاعات مورد نیاز توسط پرسشنامه ای که در خصوص تومور، علائم بالینی و اطلاعات دموگرافیک بیماران از پروندۀ بیمارانی که دارای تومور آپاندیس بودند پر شده بود ، استخراج گردید و با استفاده از آزمونهای آماری توصیفی مورد تجزیه و تحلیل قرار گرفت٬ سپس با بررسی بیماران پیش آگهی آنها مشخص گردید. یافته ها: نتایج پژوهش نشان داد که از کل مواد مورد مطالعه 5 مورد تومور آپاندیس داشتند که ازاین 5 مورد سه مورد تومور کارسنیوئید، یک مورد موکوسل و یک مورد بافت لنفاوی مرتبط با مخاط بود. علاوه بر این موارد، یک مورد کار سنیوئید کولون که به دئودنوم دست اندازی پیدا کرده و البته علائمی نیز از کار سنیوئید آپاندیس نداشت مشاهده شد. پس از مشاهده جواب پاتولوژی هیچکدام از بیمارانی که تومور کارسنیوئید آپاندیس داشتند نیاز به اقدام اضافه پیدا ننمودند ودر پی گیری 8-3 ساله علائمی از عود نداشتند. بحث و نتیجه گیری: با توجه به اینکه کارسنیوئید آپاندیس نیز می تواند بد خیم باشد ، متاستاز بدهد و باعث کاهش طول عمر بیمار گردد. دقت در تشخیص این مورد و پی گیری جواب پاتولوژی توسط بیمار و پزشک و تکمیل درمان در صورت نیاز ضروری می باشد. به کارسنیوئیدهای آپاندیس باید همانند یک نئوپلاسم نگاه کرد چرا که قدرت تهاجم موضعی و دور دست را دارد.

Title: تأثیر توام و مقایسهای مصرف ویتامین D3 و امپرازول بر جلوگیری از پیشرفت استئوپروز یائسگی در رتهای ماده

http://yafte.lums.ac.ir/article-1-175-fa.html

وقوع یائسگی و کاهش هورمونهای استروئیدی موجب بروز استئوپروز می‌گردد. درمان استئوپروز با هورمونهای استروئیدی مثل استروژن در زنان یائسه منجر به عوارض جانبی مانند سرطان پستان و سیستم تناسلی شده است. از آنجاییکه فعالیت استئوکلاستها جهت برداشت استخوان نیازمند ترشح یونهای H+ و اسیدی نمودن محیط برای جذب استخوان است. امروزه کاربرد مهارکننده‌های پمپ پروتون برای مهار فعالیت استئوکلاستها تحت بررسی است. از طرف دیگر مطالعات اخیر نشان می‌دهد که این مهارکننده‌ها با کاهش جذب کلسیم در روده، می‌توانند خطر بروز شکستگی استخوانها را افزایش دهند. با توجه به شواهد مذکور، فیزیولوژی استئوکلاستها و نحوه اثر ویتامین D‌ بر جذب کلسیم روده، در این پژوهش تأثیر توأم مصرف ویتامین D3 و امپرازول بررسی می‌گردد. مواد و روش ها: 64 رت ماده از نژاد اسپراگ با وزن تقریبی 160 تا 180 گرم انتخاب و به هشت گروه هشت تایی تقسیم شدند یک هفته بعد از عمل اورکتومی درمان گروههای سوم تا هشتم شروع و بمدت 12 هفته ادامه داشت. هنگام نمونه گیری از رتها ابتدا تحت بیهوشی خونگیری و سرم تهیه شد و سپس مهره‌های کمری و استخوانهای فیمر و تیبیای سمت راست رتها جدا و در محلول ثبوتی قرار گرفت. میزان آلکالین فسفاتاز و کلسیم سرم اندازه‌گیری شد. میزان مقاومت تنه استخوانهای فیمر در برابر شکستگی اندازه‌گیری گردید. از تنه مهره‌های L3 و انتهای استخوانهای تیبیا اسلاید بافتی تهیه گردید و تراکم حجمی تیغه‌های استخوانی تنه مهره‌های L3 نمونه‌ها با روش استریولوژیک اندازه گرفته شد. تعداد استئوکلاستها در اسلایدهای تیبیا بصورت تصادفی اندازه‌گیری شد و با استفاده از اصل ارشمیدوس چگالی مهره‌های L4 تعیین گردید.میانگین داده‌ها با نرم‌افراز Spss12 بررسی و مقایسه شدند. برای مقایسه میانگین آلکالین فسفاتاز، کلسیم سرم، چگالی مهره‌های L4 و میزان مقاومت تنه استخوانهای فیمر در برابر شکستگی از آنالیز واریانس و تست گیمزهاول و برای مقایسه میانگین تراکم حجمی تیغه‌های استخوانی و تعداد استئوکلاستها از آزمون من‌ویتنی با 0.05 < P استفاده شد. یافته ها: اورکتومی بعد از 12 هفته باعث بروز استئوپروز شدید در رتهای یائسه گروه دوم و هفتم گردید.گروههای اول (نرمال) ، سوم، چهارم، ششم و هشتم هیچگونه پوکی استخوانی را نشان ندادند و رتهای گروه پنجم بمیزان کمی دچار استئوپروز گردیدند. بحث و نتیجه گیری: مصرف ویتامین D و تا انداز‌ه زیادی DMSO از بروز و پیشرفت استئوپروز جلوگیری می‌کند. مصرف امپرازول نمی‌تواند از بروز استئوپروز جلوگیری نماید ولی در صورتیکه با دوز مناسبی از ویتامین D همراه گردد از بروز و پیشرفت استئوپروز جلوگیری می‌کند.

Title: مقایسه اثرات عصاره ساچوریاخوزستانیکا، ویتامین E ،کوآنزیم Q10 بر پراکسیداسیون LDL درمحیط In vitro

http://yafte.lums.ac.ir/article-1-176-fa.html

اکسیداسیون لیپیدها و ازجملهLDL نقش مهمی در ایجاد آترواسکلروز دارد. استفاده مواد اکسیدان در مواد غذایی باعث ایجاد LDL اکسید شده و این باعث ایجاد و پیشرفت آترواسکلروز می‌شود. بااستفاده از مواد آنتی اکسیدان مانند ویتامین E ، ساچوریاخوزستانیکا و کوآنزیم Q10 میتوان از اکسیداسیون LDL جلوگیری ودر نتیجه باعث جلوگیری از ایجاد آترواسکلروز شد. دراین مطالعهاثرات عصاره ساچوریاخوزستانیکا، ویتامین E ،کوآنزیم Q10 بر پراکسیداسیون لیپید LDLسرم درمحیط In vitro مقایسه شده است. مواد و روش ها: از افراد سالم نمونه خون تهیه شد و LDL سرم جداشده را در گروههای کنترل ، اکسید شده با مس و اکسید شده با مس وهمزمان اینکوبه باعصاره ساچوریاخوزستانیکا، ویتامین E ،کوآنزیم Q10 مطالعه شد. جهت بررسی اکسیداسیون LDL مقدار دی انهای کونجوگه و TBARS های تشکیل شده اندازه گیری شدواثرات ویتامین E ، ساچوریاخوزستانیکا و کوآنزیم Q10 برمهار اکسیداسیون LDL سرم بررسی شد. یافته ها: نتایج بدست آمده نشان میدهد که ساچوریاخوزستانیکا، ویتامین E ،کوآنزیم Q10 باعث کاهش اکسیداسیون LDL سرم می شود. بحث و نتیجه گیری: نتایج بدست آمده نشان میدهد که ساچوریاخوزستانیکا، ویتامین E ،کوآنزیم Q10 باعث جلوگیری ازاکسیداسیون LDL واین ترکیبات ممکن است اثرات مشابهی در in vivo داشته باشد.

Title: بررسی فراوانی خطاهای پزشکی درکودکان بستری شده در بیمارستان شهید مدنی خرم آباد در شش ماه اول 1387

http://yafte.lums.ac.ir/article-1-177-fa.html

بسیاری از کودکان بستری در بیمارستان از خطاهای پزشکی رنج می برند که ممکن است سبب صدمات جدی به بیماران شود. هدف از این مطالعه بررسی فراوانی خطاهای پزشکی در کودکان بستری شهید مدنی خرم آباد در شش ماه اول سال 1387 بود. مواد و روش ها: مطالعه حاضر از نوع بررسی مقطعی بود. جامعه مورد مطالعه شامل تمامی موارد خطاهای پزشکی در کودکان بستری در بیمارستان شهید مدنی شهر خرم آباد از تاریخ 1/1/87 تا 1/7/87 بود. روش نمونه گیری از نوع سرشماری بود. در این خصوص تمام پرونده ها از نظر سن، جنس، وزن، میزان تحصیلات پدر و مادر، شغل پدر و مادر، نوع خطا، خطا در چه روزی و توسط چه کسی و در چه شیفتی مورد بررسی قرار گرفتند. داده ها توسط پرسشنامه جمع آوری و با نرم افزا آماری SPSS تجزیه و تحلیل شدند. یافته ها: در این مطالعه مشخص شد که از تعداد 2250 پرونده تعداد 151 پرونده معادل 3/6 درصد دارای خطای پزشکی بودند. 53 درصد از خطاها در دخترها بود. 4/46 درصد مربوط به گروه سنی کمتر از دو سال بود. بیشتر خطای کودکان در گروه وزنی 6 کیلوگرم بود. در این مطالعه از نظر انوع خطا شامل دستور دارویی 3/46% (دوزنادرست دارو37%، فواصل دارو 28%، روش تجویز 19% وغیره 16% ) رونوشت برداری 10%، تجویزدارو 4/32% وتوزیع دارو 3/11% بود. بیشترین درصدخطا مربوط به مایع درمانی با 2/76 درصدو روش تزریقی با 4/85 درصد بود.خطا در روزهای تعطیل با تعداد 99 مورد، 6/56 درصد اتفاق افتاده بود. بیشترین درصد خطا 47 درصد مربوط به شیفت شب بود. بحث و نتیجه گیری: خطای پزشکی در بیماران بستری شایع است ودراین پژوهش3 /6 درصد دارای خطای پزشکی که باید از بروز آن جلوگیری شود.

Title: مقایسه موفقیت بی حسی نخاعی در بیماران مرد معتاد و غیر معتاد

http://yafte.lums.ac.ir/article-1-181-fa.html

مقدمه: در سالهای اخیر سوء مصرف مواد مخدر و مسأله اعتیاد، پا را از مرزهای بهداشتی و درمانی فراتر نهاده و به یک معضل اجتماعی – درمانی تبدیل شده است. از طرفی می توان بی حسی نخاعی برای انجام جراحی در اندام تحتانی برای بیمارانی که بیماریهای ریوی و بیماری سیستمیک مثل دیابت، اختلالات کبدی، اختلالات کلیوی و. . . دارند می توان به عنوان روش انتخابی استفاده کرد. در افراد معتاد هم ممکنست مشکلات ریوی هم زمان و شاید همراه مشکلات قلبی – عروقی احتمالی وجود داشته باشد. هدف از این مطالعه بررسی موفقیت بی حسی نخاعی در افراد معتاد نسبت به افراد غیر معتاد است. این مقاله استخراج شده از پایان نامه دانشجویی می باشد. مواد و روش ها: این مطالعه به صورت مقطعی انجام شده است. حجم نمونه 50 نفر بوده است که به دو گروه 25 نفری معتاد و غیر معتاد تقسیم شدند و همگی برای عمل جراحی آماده شده بودند. محدوده سنی بیماران 50-25 سال و محدوده قدی 180-165 سانتیمتر انتخاب شد. برای تایید اعتیاد یا عدم اعتیاد از تست ادراری مرفین استفاده شد. همه بیماران در وضعیت نشسته با دو سی سی لیدوکائین 5 درصد در فضای بین مهره ای L3-L4 تحت بی حسی نخاعی قرار گرفتند و پس از گذشت 5 دقیقه از انجام بی حسی، سطح بی حسی در بیماران بدون اطلاع آنان با روش Needle بررسی شد. اطلاعات لازم ثبت گردید و در نهایت داده ها توسط نرم افزار 10 SPSS مورد تجزیه و تحلیل قرار گرفت و از آزمون دقیق فیشر برای تحلیل اطلاعات استفاده شد. بحث و نتیجه گیری: بی حسی نخاعی در افراد معتاد هم بی حسی خوبی ایجاد می کند و برای بیماران کاندید جراحی روش مناسبی است. یافته ها: موفقیت بی حسی نخاعی در هر دو گروه معتاد و غیر معتاد صد درصد بود. با شروع عمل جراحی دو نفر (8%) از گروه غیر معتاد و 5 نفر‌ (20%) از گروه معتاد اظهار به درد داشتند. با بررسی مجدد سطح بی حسی در این موارد بدون اطلاع بیمار تمامی این موارد هم بی حسی داشتند که به عنوان تمارض کننده به درد در نظر گرفته شدند.

Title: اندازه گیری غلظت فلزات سنگین (آرسنیک،باریم،کادمیوم،جیوه،سرب،کرم)در منابع آب و رودخانه شهر بروجرد در سال 1378- 1388

http://yafte.lums.ac.ir/article-1-179-fa.html

همگام با رشد صنعتی و اقتصادی و تولید انواع مختلف ترکیبات و مواد شیمیایی و غیره که بشر برای رفاه و آسایش خود با استفاده از منابع طبیعی بدست آورده در این راستا بطورناخواسته موادی چون انواع فلزات سنگین و سمی را به طبیعت وارد می کند که هم برای محیط اطراف و هم برای خود مشکلات و خطرات جدی به همراه دارد. این مطالعه با هدف تعیین غلظت فلزات سنگین( آرسنیک،باریم،کادمیوم،جیوه،سرب،کرم) در کلیه چاههای تامین کننده آب شهر بروجرد وهمچنین جهت سنجش میزان آلودگی آب رودخانه شهر به این فلزات از بهمن ماه 1387تا تیر ماه 1388 انجام گرفته است. مواد و روش ها: این مطالعه از نوع بررسی (مقطعی) بوده و به منظور بررسی غلظت فلزات آرسنیک ، جیوه،کادمیوم، کروم ، سرب ، باریم در منابع آب بروجرد بصورت منظم 54 نمونه از 18 چاه تامین کننده آب شرب شهر برداشت گردید. همچنین در دو مرحله از آب رودخانه قبل و بعد ازشهر جمعا 8 نمونه برداشت گردید. پس از انتقال نمونه ها به آزمایشگاه مطابق با روش ذکر شده در کتاب استادارد متد 10 برابر تغلیظ شده و سپس عناصر مورد نظر در نمونه ها توسط دستگاه جذب اتمی (WFX 130) اندازه گیری شد و با استفاده از نرم افزار های SPSS ، Excel مورد تجزیه و تحلیل قرار گرفت. یافته ها: میانگین غلظت فلزات کادمیوم ، سرب ، کروم ، جیوه ، آرسنیک وباریم در کلیه چاههای آب شرب به ترتیب 0014/0 ، 005/0 ، 0002/0 ، 0077/0 ،0/0، 3222/0 میلی گرم بر لیتر بود. وغلظت فلزات سنگین آب رودخانه بعد از عبور از شهر افزایش چشمگیری خصوصا در فصول خشک دارد.. بحث و نتیجه گیری: نتایج بدست آمده حاکی از آن است که میانگین غلظت فلزات سنجش شده در کلیه چاههای آب شرب بروجرد پائین تر از حد استاندارد است. و هیچ اختلاف معنی داری بین غلظت فلزات در سه مرحله نمونه برداری شده مشاهده نشد (P value >0.05). نتیجه اینکه آب این چاهها آلوده به فلزا ت سنگین نیست . ولی به علت ورود فاضلابهای شهری و صنعتی به رودخانه غلظت فلزات سنگین آب رودخانه بعد از عبور از شهر افزایش زیادی داشته است و آب رودخانه را آلوده به این فلزات کرده است.

Title: بررسی میزان رضایتمندی دانشجویان از مشاوره تحصیلی اساتید راهنمای دانشگاه علوم پزشکی لرستان در نیمسال دوم سالتحصیلی 87-

http://yafte.lums.ac.ir/article-1-180-fa.html

تحقیقات انجام شده در مورد مشکلات روانی،عاطفی،اجتماعی دانشجویان اهمیت و ضرورت اقدام جدی و سامان یافته ای را در انتخاب اساتید راهنما را مطرح می نمایند. بدیهی است عدم ارائه مشاوره مناسب دستیابی دانشجویان را به اهداف آموزشی دچار اختلال می سازد . لذا با توجه به اهمیت و نقش جایگاه مشاوره و راهنمایی پژوهش حاضر با هدف تعیین میزان رضایتمندی دانشجویان از مشاوره تحصیلی اساتید راهنمای دانشگاه علوم پزشکی لرستان انجام گردید. مواد و روش ها: در این مطالعه توصیفی-مقطعی 680 دانشجوی ترم سوم و چهارم درتمام مقاطع و رشته های دانشگاه علوم لرستان به صورت سرشماری نمونه پژوهش بودند. ابزار گردآوری پرسشنامه ای بود که بر اساس دستورالعملهای وزارت بهداشت درمان و آموزش پزشکی و با ضریب پایایی 79% تهیه و نمره گذاری شد. نمرات در سه سطح رضایت دانشجویان را نشان میدادند. نتایج توسط نرم افزار spss و با روشهای آماری و آزمون کای اسکوار تجزیه و تحلیل و در سطح خطای 5% گزارش گردید. یافته ها: افراد مورد مطالعه در 9/77 % موارد زن و بین رده سنی 35-19 سال بود . بیشترین علت مراجعه به اساتید راهنما به دلیل مشکلات آموزشی (2/59%)بود. دانشجویان از اساتید راهنما در 9/37% موارد ناراضی ، 38/44% رضایت متوسط و 73/17% راضی بودند . بین دفعات مراجعه و میزان رضایت دانشجویان با ارتباط معنی دار دیده شد. بحث و نتیجه گیری: نتایج حاکی از سطح رضایتی مناسبی از اساتید راهنما نبود. لذا بایستی ضمن داشتن نگرش مثبت به برنامه مشاوره و راهنمایی تحصیلی برای اجرای آن اقداماتی نظیر اجرای قوانین ومقررات مربوط به استاد راهنما-پایش عملکرد و نتایج حاصل از فعالیت اساتید راهنما و اجرای برنامه های آموزشی مدون گام برداشت تا بتوان بهره مطلوبی را در راه ارتقاء آرمانهای دانشگاهی برداشت.

Title: بررسی فراوانی عفونت توکسو پلاسماگوندی در زنان باردار مراجعه کننده به مراکز بهداشتی درمانی شهری و روستایی الشتر در سال 87

http://yafte.lums.ac.ir/article-1-178-fa.html

مقدمه: توکسوپلاسموز یکی از شایعترین عفونتهای انگلی انسان بوده که در زنان باردار می‌تواند سقط یا ضایعات جنینی جبران‌ناپذیر ایجاد نماید. هدف این تحقیق بررسی سرولوژی عفونت توکسو پلاسما گوندی در زنان باردار مراجعه کننده به مراکز بهداشتی درمانی شهری و روستایی شهرستان الشتردر سال 87 می باشد. مواد و روش ها: تعداد 331 نفر از زنان باردار شهری و روستایی مراجعه کننده به مراکز بهداشتی درمانی شهرستان الشتر (204 نفر شهری و 127 نفر روستایی) جهت یافتن آنتی‌بادیهای اختصاصی ضد توکسوپلاسما در نمونه خون آنها با استفاده از کیتهای تجاری IgG-ELISA و IgM-ELISA تحت بررسی قرار گرفتند. سپس نتایج حاصل مورد تجزیه و تحلیل آماری قرار گرفت. بحث و نتیجه گیری: نتایج حاصل از این مطالعه نشان می‌‌دهد که افزایش سطح تحصیلات و ارتقاء آگاهی‌های بهداشتی مردم می‌‌تواند باعث کاهش میزان ابتلا به توکسوپلاسموز گردد. با توجه به اینکه 8/60% زنان باردار منطقه سابقه آلودگی به این انگل را نداشته و در معرض ابتلا به عفونت اولیه و حاد بیماری بودند، لذا اتخاذ اقدامات موثر پیشگیرانه و کنترلی کاملا ضرورت دارد. یافته ها: در این مطالعه شیوع سرمی توکسوپلاسموز در جامعه شهری و روستایی به ترتیب 2/36% و44% بود. ابتلا به عفونت مزمن توکسو پلاسموز (IgG) به ترتیب در 25% و6/34% و ابتلا به عفونت حاد توکسو پلاسموز (IgM) به ترتیب در2/11%و 4/9% زنان شهری و روستایی مشاهده گردید. بین شیوع سرمی توکسوپلاسموز (IgG و IgM) با سطح تحصیلات, سن افراد, تماس با گوشت تازه، نگهداری گربه در منزل، نوع غذا، شستن سبزیجات، نحوه مصرف سبزیجات و مصرف شیر خام در جمعیت شهری و روستایی رابطه معنی دار مشاهده گردید (P<0. 05). اما بین عیارIgG و IgM و سایر متغیرها ارتباط معنی‌داری دیده نشد.

Title: بررسی تناسب ابعاد مبلمان آموزشی و ابعاد آنتروپومتریکی دانشجویان دانشگاه علوم پزشکی اصفهان

http://yafte.lums.ac.ir/article-1-182-fa.html

اختلالات اسکلتی- عضلانی، خستگی های زودرس ناشی از فعالیت های ایستا و مواردی از این دست ریشه در طراحی نامناسب ابزار، وسایل، ساختمان ها و محیط های کاری دارد. مبلمان آموزشی با طراحی نامناسب سبب کمردرد، آسیب های کمری، دردهای ماهیچه ای، درد کتف و گردن و اختلال در گردش خون پاها می شوند. لذا ضروری است که این موضوع با دقت بیشتر مورد مطالعه قرار گیرد تا بتوان بر اساس معیارهای مدون به انتخاب این تسهیلات در مراکز آموزشی اقدام کرد. مواد و روش ها: این طرح یک مطالعه توصیفی – تحلیلی و از نوع مقطعی(Cross-sectional) می باشد. نمونه ها از میان سالن های مطالعه خوابگاه های دانشجویی و دانشکده های دانشگاه علوم پزشکی اصفهان انتخاب شد(n=150). در این مطالعه 2 ویژگی از میز و 5 ویژگی از صندلی مورد ارزیابی قرار گرفت. متغیر های مورد مطالعه با استفاده از متر فنری و گونیامتر اندازه گیری شد. نتایج حاصله با استفاده از نرم افزار SPSS 10 مورد تحلیل قرار گرفت. یافته ها: مقایسه ی میانگین مقادیر اندازه گیری شده برای ارتفاع نشیمنگاه صندلی، ارتفاع سطح رویی میزها، ارتفاع سطح زیرین میزها، عمق نشیمنگاه، پهنای نشیمنگاه و ارتفاع پشتی از سطح نشیمنگاه صندلی با مقادیر محاسبه شده در تمام موارد نشان دهنده ی اختلاف معنی داری بود (P<0.001). بحث و نتیجه گیری: با بررسی و مقایسه وضعیت مبلمان آموزشی موجود در دانشکده پزشکی دانشگاه علوم پزشکی اصفهان ارتفاع میزها زیاد و علاوه بر آن فاصله آن از تکیه گاه صندلی نامناسب است. در نتیجه دانشجو مجبور است به هنگام یادداشت مطالب بر روی میز خم شده، حمایت پشتی صندلی را از دست می دهد. همین مسئله برای افرادی که در سالن های مطالعه ی خوابگاه به مطالعه می پردازند اتفاق می افتد اما این بار به خاطر فاصله ی میز از پشتی صندلی نیست، بلکه بخاطر بلند بودن ارتفاع نشیمنگاه صندلی به وجود می آید. محققین پیشنهاد می کنند با توجه با یافته های این تحقیق، در استاندارد مربوط به مبلمان مراکز آموزشی تجدید نظر گردد.

Title: بررسی رابطه بین سن شروع دیابت نوع یک و شیوع بیماری سلیاک در کودکان و نوجوانان

http://yafte.lums.ac.ir/article-1-183-fa.html

بیماری سلیاک التهاب مزمن روده می باشد که در اثر ازدیاد حساسیت به پروتئین گلوتن ایجاد می شود. اکثر مطالعات افزایش شیوع بیماری سلیاک را در مبتلایان به دیابت نوع 1 گزارش کرده اند. هر دو بیماری منشاء اتوایمیون داشته و تحت تاثیر فاکتورهای ژنتیکی و محیطی ایجاد می شوند. هدف از این مطالعه بررسی رابطه بین شیوع سلیاک و سن ابتلا به دیابت می باشد. مواد و روش ها: در یک مطالعه توصیفی – مقطعی 135 کودک مبتلا به دیابت نوع 1 مراجعه کننده به بخش و درمانگاه غدد بیمارستان کودکان تبریز از سال 1385 تا 1387 انتخاب شدند. بعد از پر کردن مشخصات فردی بیمار و اندازه گیری قد و وزن، سطح سرمی آنتی تیشوترانس گلوتامیناز IgA آنتی بادی (A-tTG-A-IgA)، آنتی آندومیزیال IgA آنتی بادی (AEA-IgA)، آنتی گلیادین IgG آنتی بادی (AGA-IgG) اندازه گیری شد. در مواردی که مقادیر A-tTG-A یا AEA به تنهایی یا همراه با AGA بالا بود بیوپسی روده کوچک انجام شد. آنالیز داده ها با استفاده از نرم افزار spss, ver 16 انجام گردید. یافته ها: ز 135 بیمار مبتلا به دیابت نوع 1، 28 نفر از نظر سلیاک سرولوژی مثبت داشتند. شیوع بیماری سلیاک تائید شده بر اساس بیوپسی 8/6 درصد می باشد. از نظر سن شروع دیابت نوع 1 و ابتلا به بیماری سلیاک رابطه آماری معنی داری مشاهده نشد (996/0=P). بحث و نتیجه گیری: بیماری سلیاک در افراد مبتلا به دیابت نوع 1 رابطه ای با سن شروع دیابت ندارد و پیگیری بیماران مبتلا به دیابت نوع 1 از نظر بیماری سلیاک باید در طول دوره درمان و پیشگیری انجام شود.

Title: تاثیر فعالیت بدنی با شدت مساوی و هزینه انرژی متفاوت برچربی و لیپوپروتئین های خون مردان با چربی خون بالا

http://yafte.lums.ac.ir/article-1-194-fa.html

هایپرلپیدمی یا افزایش میزان چربی های خون، وضعیتی است که به خصوص در طی دهه های اخیر به عنوان یک بیماری مهم، شیوع آن افزایش یافته است. با توجه به مشکلات مرتبط با مصرف دارو و همچنین فواید فعالیت بدنی در بهبود هایپرلپیدمی، در این تحقیق سعی شده است که به بررسی تاثیر فعالیت بدنی با توجه به میزان هزینه انرژی بر لیپوپروتئین های خون پرداخته شود. مواد و روش ها: بدین منظور تعداد 21 نفر مرد مبتلا به هایپرلپیدمی باتوجه به شرایط تحقیق انتخاب شده و پروتکل تمرینی را اجرا کردند (فعالیت بدنی با هزینه انرژی 450 و 600 کیلوکالری). یافته ها: نتایج به دست آمده پس از 8 هفته تمرین نشان داد که سوزاندن 450 کیلوکالری در هر جلسه باعث تغییر معنادار در سطوح کلسترول توتال و HDL می شود(P≤0/05). با این حال برای ایجاد تغییر معنادار در سطوح تری گلیسیرید به سوزاندن 600 کیلوکالری در جلسه نیاز است(P≤0/05). میزان سطوح LDL در هر دو گروه تحقیق تغییر معناداری نداشت و می توان نتیجه گرفت که سوزاندن 600 کیلوکالری در جلسه برای ایجاد تغییر در سطوح LDL کافی نمی باشد. بحث و نتیجه گیری: به طور کلی می توان نتیجه گرفت که به منظور ایجاد تغییرات مثبت در سطوح HDL می توان از فعالیت کمتری سود جست ولی برای ایجاد تغییرات مثبت در سطوح تری گلیسیرید می بایست میزان هزینه انرژی فعالیت را بیشتر کرد.

Title: تأثیر خود مراقبتی بر کیفیت زندگی مبتلایان سکته مغزی

http://yafte.lums.ac.ir/article-1-195-fa.html

یکی از اختلالات نورولوژیکی که عملکرد قسمتی از بدن و درک فیزیکی از وضعیت بدن را تغییر می دهد؛ سکته مغزی است و از آنجا که بر اساس آمارهای رایج سکته مغزی در جایگاه سوم علل طبی مرگ در کشورهای پیشرفته است از اهمیت به سزایی برخوردار است. خودمراقبتی یکی از ابعاد بازتوانی مبتلایان سکته مغزی محسوب می شود. با حمایت پرستار از خودمراقبتی و انجام آن توسط بیمار، وی کنترل خود را دوباره به دست آورده و احساس ارزش نفس می نماید. مواد و روش ها: این مطالعه بر روی 42 بیمار مبتلا به سکته مغزی انجام شد. ابزار گردآوری داده ها پرسشنامه استاندارد اختصاصی سنجش کیفیت زندگی پس از سکته مغزی(SS-QOL) بود. در ابتدا شاخص کیفیت زندگی در هر دو گروه مطالعه و شاهد اندازه گیری شد، سپس در گروه مطالعه برنامه خود مراقبتی آموزش و سپس به مدت 5 هفته اجرا شد. در پایان 5 هفته مجدداً شاخص کیفیت زندگی در هر دو گروه سنجیده و مقایسه گردید. یافته ها: تفاوت آماری معنی داری بین کیفیت زندگی قبل و پس از انجام خود مراقبتی در گروه مورد مشاهده شد (001/0

Title: بررسی نیازهای بهداشت روانی مردم شهر همدان

http://yafte.lums.ac.ir/article-1-196-fa.html

برای تمامی افراد، سلامت روانی، فیزیکی و اجتماعی اجزای ضروری حیات هستند و به شدت بهم وابسته اند و سلامت روان در رفاه کلی افراد، جوامع و کشورها نقش اساسی دارد. هدف از این مطالعه تعیین نیازهای بهداشت روانی مردم شهر همدان می باشد. مواد و روش ها: این پژوهش یک مطالعه توصیفی – مقطعی است و حجم نمونه شامل 1300 نفربود که با روش نمونه گیری طبقه ای انتخاب گردیدند.، ابزار گرد آوری داده ها پرسشنامه و شامل سه بخش الف- مشخصات دموگرافیک ب- سوالات مربوط به نحوه ارائه خدمات بهداشت روانی ج – سوالات مربوط به نیازهای بهداشت روانی بود. روش جمع آوری داده ها از طریق مصاحبه سازمان یافته انجام گرفت. یافته ها: یافته های حاصل نشان داد که ارائه خدمات بهداشت روانی از دیدگاه مردم شهر همدان ضعیف بوده و اکثرا معتقد بودند در محل زندگی آنها دسترسی آسان به خدمات بهداشت روانی وجود ندارد (76%) و برنامه های آموزشی در مورد بهداشت روان توسط رسانه های گروهی محلی ضعیف است (34%). همچنین اکثرا آموزش بهداشت روان به گروههای مختلف جمعیتی (72%) ، ایجاد مراکز بهداشت روان در مدارس(8/52%)، کارخانجات (7/50)، گسترش مراکزجامع روانپزشکی (8/575)، گسترش خدمات بیمه ای در خصوص تامین هزینه های بهداشت روانی (6/85%)، اصلاح باورهای غلط در مورد بیماری روانی(6/42%) و اهمیت دادن به سلامت روانی (9/50%) را از نیازهای بهداشت روانی در شهر همدان بیان نمودند بحث و نتیجه گیری: خدمات سلامت روانی مبتنی بر جامعه باید درمان ها و مراقبت های محلی را ارایه نمایند. این خدمات باید جامع باشند، به نحوی که بتوانند طیفی از تسهیلات را برای رسیدگی به نیازهای سلامت روان کل جمعیت و نیز گروههای خاص از قبیل کودکان، نوجوانان، زنان و سالمندان تامین کنند.
